# Effects of different extrusion temperatures on extrusion behavior, phenolic acids, antioxidant activity, anthocyanins and phytosterols of black rice†

**DOI:** 10.1039/c7ra13329d

**Published:** 2018-02-14

**Authors:** Zhanqiang Hu, Xiaozhi Tang, Ming Zhang, Xianqiao Hu, Chen Yu, Zhiwei Zhu, Yafang. Shao

**Affiliations:** Rice Product Quality Supervision and Inspection Center, Ministry of Agriculture, China National Rice Research Institute Hangzhou 310006 China hzq8362029@163.com yafang_shao@126.com +86 571 63370380 +86 571 63370351; College of Food Science and Engineering, Nanjing University of Finance and Economics Nanjing 210046 China; Jiangsu Grain and Oil Commodity Trade Market Nanjing 210003 China

## Abstract

Different extrusion temperatures (90, 100, 110, and 120 °C) were used to investigate changes in the expansion ratios, die pressures, phytochemical contents and antioxidant activities of extrusion products of black rice. The results showed that the die pressure significantly decreased with the increasing extrusion temperature, and the expansion ratio reached a peak value at 100 °C. The soluble-free and total phenolic acid contents gradually increased, whereas portions of soluble-free and soluble-conjugated phenolic acids transformed into insoluble-bound phenolic acids. The soluble-free (52.45) and insoluble-bound (73.59 mg GAE/100 g DF) total phenolic contents (TPC) reached peak values at 110 °C. The soluble-conjugated TPC values remained similar. Antioxidant activity occurred at higher levels in the range from 100 °C to 120 °C. The anthocyanin content decreased after extrusion possibly because some anthocyanin remained in the residue after extraction and could not be completely extracted. The content of free sterols increased from 90 °C to 110 °C and decreased at 120 °C. However, the content of bound sterols showed an opposite trend and reached a minimum value at 110 °C.

## Abstract 2

Different extrusion temperatures (90, 100, 110, and 120 °C) were used to investigate changes in the expansion ratios, die pressures, phytochemical contents and antioxidant activities of extrusion products of black rice.

## Graphical abstract

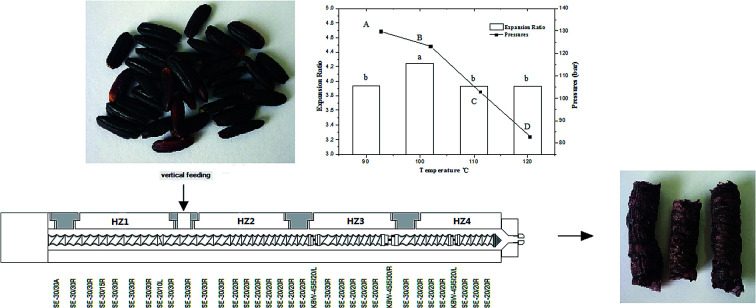

## 1. Introduction

Rice is a staple food for almost half the world's population and is cultivated in over 100 countries. Whole grain rice de-husked from rice, is a typical whole grain that plays an important role in good health and a balanced diet; thus, it is becoming popular in western countries and is being more gradually accepted in developing countries.^1^ Black rice is a special type of whole grain rice. It is well known that the pericarp (outer part) of kernels of this rice is black due to a pigment known as anthocyanin. Anthocyanin is an antioxidant, which has been shown to have highly beneficial contribution to nutritional and therapeutic values as compared to milled white rice. Therefore, black rice is also known as heaven rice, imperial rice, king's rice, and prized rice because of its functional effects and edible history.^2^ Many interventional and epidemiological studies have reported that black rice is a good source of phytochemicals, fiber, and minerals as well as basic nutrients;^3^ moreover, the regular consumption of black rice can reduce the risk of chronic diseases such as cardiovascular diseases, type II diabetes, obesity and some cancers.^4,5^

Extrusion is a thermal process that mainly involves the application of mixing, high heat, high pressure, shear forces, drying, and partial texturing to an uncooked mass. Due to its advantages of low cost, short time, high productivity, versatility, unique product shapes and energy efficiency, extrusion has been popular since the mid 1930s. In the past decade, extrusion technology has been used extensively to produce snake foods, baby foods, breakfast cereals, and pet foods because this process cannot only improve digestibility and nutrient bioavailability, but can also be used to develop a range of products with distinct textural properties including crispiness, expansion, and mouthfeel.^6^ In food extrusion processing, various parameters, such as extrusion temperature, screw speed and the moisture content of the material, play important roles in retaining functional compounds and improving textural properties; these parameters can affect the extrusion responses, which can also influence the quality of the extrusion products. Wang *et al.* found a negative linear correlation between extrusion temperature and die pressure, and the increase of screw speed resulted in the decrease of die pressure and motor torque.^7^

Whole grain rice is considered to be a desirable processing material for extrusion due to its abundant phytochemical components and fiber content, hypoallergencity, bland taste and ease of digestion.^8^ Many studies have been performed to investigate the effects of extrusion on the phytochemical content and textural properties of whole grain rice and optimize the controlling parameters to obtain extrusion products with rich nutritional compounds and good textural properties.^9^ Ti *et al.* showed that extrusion at 120 °C decreased the free, bound phenolics and anthocyanins of black rice.^10^ However, it has been reported that the same extrusion temperature can increase bound phenolic and soluble dietary fiber contents and improve antioxidant properties.^11,12^ Brennan *et al.* reported that extrusion technology may improve the bioavailability of these bioactive compounds by forming complexes with proteins which can be broken down in the human body, thus yielding antioxidant activity.^6^ However, high extrusion temperatures (over 120 °C) can significantly decrease the phytochemical content and antioxidant activity of whole grain rice.^13^ Sarawong *et al.* also reported that a high extrusion temperature of 130 °C resulted in decreases of the total phenolic content and antioxidant activities of free and bound phenolics.^14^ Little information has been reported about the effects of different extrusion temperatures (below 120 °C) on the expansion ratios, die pressures, phenolic acids, antioxidant activities, anthocyanins and sterol contents of extrusion products of black rice. Therefore, in this work, black rice was used as an experimental material and subjected to different extrusion temperatures (including 90 °C, 100 °C, 110 °C and 120 °C). Our aim was to investigate the effects of different extrusion temperatures on the textural and functional properties of rice products based on whole grain rice and provide useful information for the food industry.

## 2. Materials and methods

### 2.1 Samples and chemistry reagents

Black rice (*Oryza sativa* var. Pinhei 01) was obtained from the Anhui Academy of Agricultural Science in 2015. The rice grains were de-husked and ground with a mill (Foss, Switzerland); all sample flours were sieved by passing through a 60-mesh sieve and were stored at 4 °C in the dark in a tight plastic bag prior to analysis. The moisture content was determined by drying at 105 °C to a constant weight. The analytical results are expressed on a dry matter basis.

HPLC-grade methanol, ethyl acetate ethanol, *n*-hexane and diethyl ether were purchased from Merck (Darmstadt, Germany) and Tedia (Fairfield, USA), respectively. Phenolic acid, anthocyanin and sterol standards were purchased form Sigma-Aldrich Chemical Co. (St. Louis, MO, USA). Analytical grade sodium hydroxide, sodium sulfate, and hydrochloric acid were purchased from Sinopharm Chemical Reagent Co., Ltd (Shanghai, China). Folin–Ciocalteu reagent, sodium hydroxide, DPPH (2,2-diphenyl-1-picrylhydrazyl), trolox (6-hydroxyl-2,5,7,8-tetramethylchroman-2-carboxylic acid), and ABTS˙^+^ (2,2ʹ-azinobis-(3-ethylbenzene-hiazoline-6-sulfonic acid) diammonium salt) were purchased from Sigma-Aldrich Chemical Co. (St. Louis, MO, USA).

### 2.2 Extrusion

The extrusion process was performed using a co-rotating twin-screw extruder (Brabender DSE 20/40, Duisburg, Germany) with a length-to-diameter ratio of 40 and a screw diameter of 20 mm, containing four independent zones (HZ1, HZ2, HZ3 and HZ4) of controlled temperature in the barrel. The diameter of the hole in the die was 4 mm, with a die length of 15 mm. The black rice flour was fed to the extruder using a gravimetric feeder. The feed and compression metering zones were kept constant at 40 °C (HZ1), 60 °C (HZ2) and 90 °C (HZ3), respectively, and the die head and HZ4 temperatures underwent synchronized changes at 90 °C, 100 °C, 110 °C and 120 °C, respectively. The feeding screw and extruder screw were run at constant speeds of 12 and 150 rpm, respectively. When stable conditions were established, the extrudates were collected and dried using an air oven at 40 °C for 24 h. The diameters of 10 pieces of extrudates were measured by a Vernier calliper, and the expansion ratio was calculated as the ratio of the diameter of the extrudate to the diameter of the die hole. The die pressure was measured with a pressure transducer directly in front of the orifice. Readings were recorded every 20 s for at least 4 min, and the average values were expressed as bars. Black rice and all extrudates were ground with a mill (Foss, Switzerland) and passed through a 60 mesh sieve. The black rice was marked as BR; the samples extruded at 90 °C, 100 °C, 110 °C and 120 °C were marked as T90, T100, T110, and T120, respectively. All flour samples were finally placed in plastic bags and were stored at −4 °C prior to analysis.

### 2.3 Extraction of soluble-free, soluble-conjugated and insoluble-bound phenolics

Extraction of the soluble-free phenolics in black rice and its extrusion products was performed according to a previously described method with minor modifications.^10,15^ For example, 0.5 g sample flour was extracted twice with 20 ml chilled acidified methanol (95% methanol and 1 M HCl, 85 : 15 v/v). Each time, the mixture was shaken and centrifuged. The supernatants were collected and combined. After adjusting the pH to about 1.5 to 2.0 with concentrated HCl, the supernatant was concentrated, defatted and then extracted three times using ethyl acetate (70 ml). The ethyl acetate extracts were pooled and rotary evaporated at 35 °C. The dried extracts were dissolved in 5 ml 50% methanol as soluble-free phenolic extracts. All analyses were performed in triplicate.

Extractions of soluble-conjugated phenolics in black rice and its extrusion products were performed according to the method reported by Li, Shewry, and Ward with minor modifications.^16^ In short, 0.5 g sample flour was extracted twice with 20 ml chilled acidified methanol (95% methanol and 1 M HCl, 85 : 15 v/v); after shaking and centrifugation, the supernatants were collected and combined. The concentrated supernatants were hydrolyzed with 4 M NaOH (40 ml) for 2 h under nitrogen, and then the mixture was adjusted to a pH of about 1.5 to 2.0 with concentrated HCl. The solution was defatted and extracted with ethyl acetate by following the procedure described above. Finally, the dried extracts were dissolved in 5 ml 50% methanol as soluble-conjugated phenolic extracts. All analyses were performed in triplicate.

After extracting the soluble-free phenolics or soluble-conjugated phenolics, the residue was washed with distilled water and added to 4 M NaOH (40 ml) to extract the insoluble-bound phenolics. After shaking for 2 h at room temperature, the mixture was adjusted to pH 1.5 to 2.0 with concentrated HCl. After centrifugation at 10 000 × *g* for 30 min at 4 °C, the supernatant was collected, defatted and extracted with ethyl acetate. The ethyl acetate fractions were combined and evaporated at 35 °C. The dried extracts were dissolved in 5 ml 50% methanol and used as insoluble-bound phenolic extracts. All analyses were performed in triplicate.

### 2.4 HPLC analysis of phenolic acids

For HPLC analysis, the soluble-free, soluble-conjugated and insoluble-bound phenolic extracts were filtered through 0.45 μm membrane filters before analysis. Then, 10 μl of each sample extract was fractionated using a waters HPLC system equipped with a waters 2707 autosampler, a Waters 2489 UV/Visible detector and a Waters 1525 binary pump on a 250 mm × 4.6 mm i.d., 5 μm, Inertsil C18 analytical column. Gradient elution was performed with a mobile phase consisting of A (0.1% acetic acid in water) and B (0.1% acetic acid in methanol). The flow rate was 1 ml min^−1^, and the gradient elution was set according to the method reported by Shao *et al.* as follows: 0 to 11 min, 9% to 14% B; 11 to 14 min, 14% to 15% B; 14 to 17 min, 15% B; 17 to 24 min, 15% to 16.5% B; 24 to 28 min, 16.5% to 19% B; 28 to 30 min, 19% to 25% B; 30 to 36 min, 25% to 26% B; 36 to 38 min, 26% to 28% B; 38 to 41 min, 28% to 35% B; 41 to 46 min, 35% to 40% B; 46 to 48 min, 40% to 48% B; 48 to 53 min, 48% to 53% B; 53 to 70 min, 53% to 70% B; 70 to 72 min, 9% B; 72 to 75 min, 9% B.^15^ The column was warmed to 40 °C. The phenolic acids were detected at a wavelength of 280 nm, and the phenolic acid contents were quantified using the external calibration curves.

### 2.5 HPLC analysis of anthocyanins

Extraction and determination of anthocyanins were performed using the methods previously reported by Ti *et al.* and Shao *et al.* with minor modifications.^10,15^ Briefly, 0.5 g sample flour was extracted twice with 10 ml chilled acidified methanol (95% methanol and 1 M HCl, 85 : 15 v/v). Each time, the mixture was shaken on a shaker (HY-8 Speed control shaker, China) for 30 min at room temperature and then centrifuged (Himac CR21GII, Hitachi, Japan) at 4000 rpm for 15 min. The supernatants were collected and combined. The concentrated extracts were filtered through 0.45 μm membrane filters before analysis. The HPLC system described above was also used for anthocyanin analysis. The gradient elution was conducted with 0.5% formic acid in water (solution A) and 0.5% formic acid in methanol (solution B) at a flow rate of 0.5 ml min^−1^. The gradient was set as follows: 0 to 5 min, 10% B; 5 to 8 min, 10% to 15% B; 8 to 13 min, 15% to 20% B; 13 to 18 min, 20% to 25% B; 18 to 25 min, 25% to 30% B; 25 to 30 min, 30% to 35% B; 30 to 35 min, 35% to 50% B; 35 to 40 min, 50% to 60% B; 40 to 45 min, 60% to 95% B; 45 to 50 min, 95% to 10% B; 50 to 58 min, 10% B. The UV detection was set at a wavelength of 520 nm. The identification and quantification of anthocyanins was based on their retention times and chromatography of the standards.

### 2.6 Determination of total phenolic content (TPC)

The Folin–Ciocalteu method was used to measure the TPC of black rice and its extrusion products, with minor modifications.^17^ Briefly, 200 μl of appropriate diluted soluble-free, soluble-conjugated, or insoluble-bound phenolic extracts or standard solutions were added to 1.5 ml of 10-fold diluted Folin–Ciocalteu reagent and then neutralized with 1.5 ml saturated sodium carbonate (75 g l^−1^). After incubating for 2 h in the dark, the absorbance of the mixture was measured at 725 nm by a spectrophotometer (Unico Co, USA). Gallic acid solution was prepared and used as a standard to generate the straight line equation. The TPC results were expressed as milligrams of gallic acid equivalent (GAE) per 100 g of rice flour on a dry matter basis (mg GAE/100 g DF). Duplicate determinations were carried out for each extract.

### 2.7 Determination of DPPH radical scavenging activity (DPPH)

The DPPH assay was performed with slight modifications.^18^ Soluble-free, soluble-conjugated, or insoluble-bound phenolic extracts or standards (200 μl) were added to 100 μmol l^−1^ DPPH solution (3 ml). After incubation for 30 min in the dark, the absorbance was measured at the wavelength of 517 nm. The DPPH radical scavenging activity (%) was calculated using the following formula:DPPH (%) = (1 − *A*_sample_/*A*_control_) × 100%

According to the straight line equation of the Trolox standard, the results were expressed as μmol of Trolox equivalent DPPH radical scavenging activity per 100 g of rice flour on a dry matter basis (μM TE/100 g DF). Duplicate determinations were carried out for each extract.

### 2.8 Determination of ABTS˙^+^ radical scavenging activity (ABTS)

The measure of ABTS˙^+^ radical scavenging activity was carried out according to the method previously reported by Shen *et al.*^17^ The results were calculated according to the straight line equation of the Trolox standard and expressed as μmol of Trolox equivalent activity per 100 g of rice flour on a dry matter basis (μM TE/100 g DF). Duplicate determinations were carried out for each extract.

### 2.9 Extraction and analysis of free and bound sterol fractions

Lipid extraction was performed using a Soxhlet extractor. Appropriately 6 g sample flour was extracted with 30 ml of petroleum ether for 6 h. The extracts were collected and dried under a stream of nitrogen. The lipid extract was weighed and stored at 4 °C prior to analysis.

The sample preparation protocols were performed using the method reported by Hu *et al.*^19^ In brief, the lipid extract was dissolved in 5 ml of *n*-hexane; 100 μl of 5 μg ml^−1^ 5α-cholestanol was added as an internal standard, and the mixture was then applied to silica SPE cartridges (ProElut Silica 500 mg/6 ml, Dilma, China) previously equilibrated with 5 ml of *n*-hexane. The less polar compounds were eluted with 10 ml of *n*-hexane/diethyl ether (95 : 5 v/v). The retained sterols and SOPs were eluted using a highly polar solvent system with 10 ml of *n*-hexane/diethyl ether (80 : 20 v/v). Finally, the free sterol extract was dried under a stream of nitrogen and stored at 4 °C prior to analysis.

The bound sterol could not be directly measured, but could be calculated by the difference between the total sterol and free sterol contents. Cold saponification was used to determine the content of total sterol. The method described by Azadmard-Damirchi and Dutta^20^ was performed with minor modifications. In brief, the lipid extract was blended with 100 μl of 5 μg ml^−1^ 5α-cholestanol and 10 ml of KOH (1 M) in 95% ethanol. The mixture was shaken on a vortex shaker for 1 min and incubated at 70 °C for 45 min before cooling to room temperature. 5 ml of dichloromethane and 3 ml distilled water were added, and then the mixture was shaken vigorously to extract the unsaponifiable fraction containing the SOPs along with unoxidized sterols. Finally, the aqueous phase was removed and the organic phase was washed repeatedly with distilled water until the solution became clear. The organic phase was dried under a stream of nitrogen and stored at 4 °C prior to analysis.

The dried extract and standard solutions were derivatized with 100 ml of derivatives (*N*-methyl-*N*-(trimethylsilyl) heptafluor-obutyramide and 1-methylimidazole, 95 : 5, v/v) at 75 °C for 20 min; after cooling to room temperature, the mixture was diluted with 1 ml of *n*-hexane and then injected into a gas chromatograph. A GC-MS system consisting of a 7683 auto-sample injector, 6890 GC-System and 5973 Mass Selective detector was used for sterol analysis. An arylene type 5% phenyl-95% methyl polysiloxane fused silica capillary column, DB-5MS (30 m × 0.25 mm × 0.25 mm, Agilent Technologies, USA) was used for separation. Chromatographic and mass spectrometer conditions were performed using the method previously described by Hu *et al.* with minor modifications as follows: helium carrier gas was used at a flow rate of 1.2 ml min^−1^. The oven temperature was initially set at 100 °C for 1 min and then gradually increased to 290 °C at a rate of 40 °C min^−1^. Hot splitless injection was performed at 300 °C. The ion source temperature was set at 250 °C and the transfer line was set at 300 °C. Identification was supported by comparison with the mass spectrum of trimethylsilylation of a standard substance. Quantification was performed by the addition of an internal standard and by using the straight line equation of the standard. Selective ion monitoring (SIM) was used for identification and quantification of the compounds. The results were expressed using two units as mg per 100 g rice flour on a dry matter basis (mg/100 g DF) and mg per 1 g extracted oil (mg g^−1^ oil). The measurements were performed using the method reported by Hu *et al.*^19^ The results were expressed using two units as mg per 100 g rice flour on a dry matter basis (mg/100 g DF) and mg per 1 g extracted oil (mg g^−1^ oil).

### 2.10 Statistical analysis

All procedures were carried out with at least three replications and are presented on a dry weight basis as mean ± standard deviation (SD). Data analysis was performed using the SAS program version 8 (SAS Institute Inc., Cary, NC, U.S.A.). Correlation analysis between the extrusion temperature, die pressure and various parameters (phenolics, TPC, DPPH and ABTS) was performed with Pearson's correlation test. The significance of the differences between different extrusion temperatures was determined using ANOVA, followed by Duncan multiple comparison tests. Statistical significance was defined at a level of *P* < 0.05.

## 3. Results and discussion

### 3.1 Effects of different extrusion temperatures on the expansion ratios and die pressures of the extrudates

**Fig. 1:**
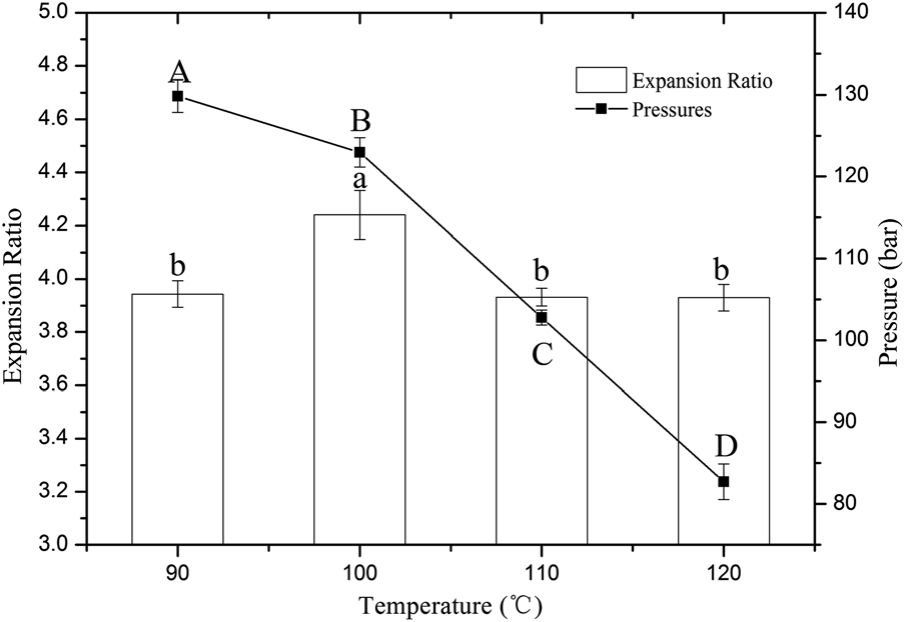
Effects of different extrusion temperatures on the expansion ratios and die pressures of the extrudates. *Different lowercase and capital letters above the bars indicate significant differences (*P* < 0.05).

The changes in the die pressures and expansion ratios of the extrudates are shown in Fig. 1. The results indicate that the die pressure significantly (*P* < 0.05) decreased, with a slight reduction from 90 °C to 100 °C and a large decline from 100 °C to 120 °C. In extrusion processing, starch granules under high temperature begin to swell, and amylose diffuses out of the granules with uncoiling of the double helices until the crystallites are almost completely disrupted and form a viscous molten gel.^21^ However, lower temperatures and short extrusion times can result in incomplete melting of the starch granules, resulting in a higher viscosity of the molten gel and higher die pressure. Akdogan *et al.* reported that raw materials require longer heating times to form a viscoelastic fluid at low temperatures.^22^ When the extrusion temperature was over 100 °C, the die pressure significantly decreased as the temperature increased, which is consistent with a previous report by Wang *et al.*^7^ This may be due to the viscoelastic fluid passing heat into a free flowing fluid due to overheating.^7^ When the extrudate exits from the die orifice, it begins to expand because the water under high pressure is suddenly released. The expansion ratio can reflect the degree of expansion, which mainly depends on higher die pressure and vapor pressure. In this study, the expansion ratios of the extrudates reached a peak value at 100 °C and showed no significant difference at 90 °C, 110 °C or 120 °C. As mentioned above, lower temperature resulted in higher die pressure but led to a decrease in vapor pressure, which is a major driving force for expansion. Hence, the expansion ratio was lower at 90 °C. Similar trends were observed in previous studies.^23,24^ When the extrusion temperature was over 100 °C, the die pressure significantly decreased, which also resulted in a decrease of the extrusion ratio. Therefore, the interaction between the extrusion temperature and die pressure resulted in a peak value of the expansion ratio of the extrudates at 100 °C.

### 3.2 Effects of different extrusion temperatures on phenolic composition

Phenolic acid is an important source of antioxidant activity and has two forms in rice: the soluble form, including soluble-free and soluble-conjugated forms; and the insoluble-bound form, where the acids are esterified to cell walls.^15^ Ferulic and *p*-coumaric acid have been observed to be the most abundant phenolic acids in black rice. As shown in Table 1, ten phenolic acids were identified in the soluble-free, soluble-conjugated and insoluble-bound phenolic acid fractions, including gallic acid (GA), protocatechuic acid (PA), 2,5-dihydroxybenzoic acid (2,5-HA), *p*-hydroxybenzoic acid (*p*-HA), vanillic acid (VA), sinapic acid (SA), *p*-coumaric acid (*p*-CA), ferulic acid (FA), sinapic acid (SNA) and isoferulic acid (IFA).

**Table 1:** Changes in soluble-free, soluble-conjugated and insoluble-bound phenolic acids of extrudates at different extrusion temperatures^a^

Phenolic	Treatment	Soluble-free	Soluble-conjugated	Insoluble-bound	Total
GA	BR	4.34 ± 0.99^b^	41.32 ± 0.52^a^	1.40 ± 0.34^b^	42.72 ± 0.38^a^
	T90	4.97 ± 0.47^b^	19.13 ± 0.79^c^	2.62 ± 0.46^a^	21.75 ± 0.46^c^
	T100	8.12 ± 0.42^a^	21.06 ± 0.69^bc^	2.86 ± 0.33^a^	23.91 ± 0.53^b^
	T110	7.73 ± 0.63^a^	22.36 ± 0.81^b^	2.58 ± 0.09^a^	24.94 ± 0.85^b^
	T120	8.47 ± 0.14^a^	20.91 ± 2.05^bc^	3.09 ± 0.16^a^	24.00 ± 1.89^b^
PA	BR	7.30 ± 1.31^c^	18.28 ± 0.39^a^	11.67 ± 0.17^c^	29.95 ± 1.36^c^
	T90	9.25 ± 0.25^b^	11.45 ± 0.02^bc^	27.62 ± 0.04^ab^	39.07 ± 0.38^b^
	T100	9.99 ± 0.83^ab^	13.06 ± 0.40^b^	25.54 ± 0.29^b^	38.60 ± 2.46^b^
	T110	10.83 ± 0.02^a^	12.37 ± 0.68^bc^	32.18 ± 0.26^a^	44.56 ± 3.24^a^
	T120	10.99 ± 0.19^a^	10.47 ± 1.81^c^	29.70 ± 0.06^ab^	40.17 ± 1.25^ab^
2,5-HA	BR	10.34 ± 0.10^a^	14.70 ± 1.59^a^	ND	14.70 ± 1.59^b^
	T90	4.53 ± 0.59^d^	8.87 ± 0.18^b^	12.22 ± 0.07^b^	21.09 ± 0.26^a^
	T100	5.31 ± 0.28^cd^	5.35 ± 0.19^c^	12.25 ± 1.58^b^	17.60 ± 1.39^b^
	T110	6.15 ± 0.60^cd^	7.41 ± 0.04^bc^	13.36 ± 0.41^ab^	20.77 ± 0.45^a^
	T120	7.08 ± 0.77^b^	7.97 ± 1.42^b^	14.96 ± 0.18^a^	22.93 ± 1.60^a^
*p*-HA	BR	1.07 ± 0.28^a^	4.41 ± 0.08^a^	1.61 ± 0.01^b^	6.01 ± 0.12^b^
	T90	0.83 ± 0.08^a^	2.24 ± 0.09^b^	5.10 ± 0.15^a^	7.33 ± 0.28^a^
	T100	0.92 ± 0.16^a^	2.35 ± 0.09^b^	4.91 ± 0.89^a^	7.22 ± 0.81^a^
	T110	0.95 ± 0.09^a^	2.25 ± 0.11^b^	5.18 ± 0.36^a^	7.47 ± 0.47^a^
	T120	1.04 ± 0.05^a^	2.02 ± 0.10^c^	5.13 ± 0.18^a^	7.21 ± 0.22^a^
VA	BR	11.41 ± 2.68^d^	115.01 ± 5.47^a^	33.49 ± 4.59^d^	148.50 ± 9.13^ab^
	T90	20.52 ± 0.15^c^	62.83 ± 3.20^b^	78.81 ± 1.83^c^	141.65 ± 4.25^b^
	T100	22.21 ± 0.41^bc^	67.69 ± 2.82^b^	88.89 ± 0.34^b^	156.58 ± 2.50^a^
	T110	23.17 ± 0.28^ab^	67.39 ± 4.19^b^	86.69 ± 2.01^b^	154.08 ± 5.75^a^
	T120	25.08 ± 0.07^a^	61.94 ± 3.70^b^	96.53 ± 1.38^a^	158.46 ± 3.05^a^
SA	BR	TR	2.70 ± 0.07^a^	ND	2.70 ± 0.07^b^
	T90	TR	1.54 ± 0.04^b^	2.24 ± 0.02^bc^	3.78 ± 0.05^a^
	T100	TR	1.47 ± 0.12^b^	2.27 ± 0.10^ab^	3.74 ± 0.17^a^
	T110	TR	1.45 ± 0.09^b^	2.23 ± 0.04^c^	3.68 ± 0.11^a^
	T120	TR	1.36 ± 0.18^b^	2.38 ± 0.03^a^	3.74 ± 0.15^a^
*p*-CA	BR	1.68 ± 0.13^c^	5.41 ± 0.13^a^	15.58 ± 2.48^b^	20.99 ± 2.43^b^
	T90	1.78 ± 0.02^bc^	3.43 ± 0.13^b^	18.58 ± 0.24^a^	22.01 ± 0.34^ab^
	T100	1.86 ± 0.03^b^	3.83 ± 0.09^b^	19.01 ± 0.89^a^	22.84 ± 0.91^ab^
	T110	1.86 ± 0.03^b^	3.59 ± 0.41^b^	19.31 ± 0.61^b^	22.90 ± 0.85^ab^
	T120	2.01 ± 0.01^a^	3.35 ± 0.38^b^	20.12 ± 0.23^a^	23.47 ± 0.36^a^
FA	BR	6.62 ± 0.90^a^	29.99 ± 2.14^a^	222.50 ± 3.15^c^	251.35 ± 4.25^b^
	T90	4.47 ± 0.13^b^	15.72 ± 0.90^b^	235.14 ± 1.72^b^	251.14 ± 2.79^b^
	T100	4.41 ± 0.12^b^	18.09 ± 0.91^b^	250.07 ± 3.08^a^	267.69 ± 2.48^a^
	T110	3.85 ± 0.94^b^	16.53 ± 1.71^b^	250.59 ± 3.58^a^	268.03 ± 2.68^a^
	T120	3.98 ± 0.41^b^	16.01 ± 2.18^b^	257.16 ± 0.87^a^	274.34 ± 1.98^a^
SNA	BR	2.36 ± 0.07^ab^	20.75 ± 1.31^a^	ND	20.75 ± 1.31^a^
	T90	1.33 ± 0.01^b^	4.43 ± 0.36^d^	9.33 ± 1.01^a^	13.77 ± 1.37^b^
	T100	2.71 ± 0.15^a^	4.69 ± 0.08^cd^	9.79 ± 0.31^a^	14.47 ± 0.39^b^
	T110	2.25 ± 0.88^ab^	6.78 ± 0.61^b^	8.50 ± 0.24^a^	15.29 ± 0.37^b^
	T120	2.62 ± 0.25^a^	6.25 ± 0.08^bc^	8.18 ± 1.35^a^	14.43 ± 1.44^b^
IFA	BR	1.26 ± 0.75^b^	6.66 ± 0.16^a^	19.27 ± 3.31^a^	26.74 ± 4.41^a^
	T90	2.15 ± 0.68^ab^	4.33 ± 0.32^b^	18.60 ± 0.69^ab^	22.99 ± 1.29^ab^
	T100	3.22 ± 0.17^a^	2.42 ± 0.12^d^	15.52 ± 1.47^bc^	17.72 ± 2.14^bc^
	T110	3.55 ± 0.01^a^	3.27 ± 0.23 ^cd^	14.02 ± 0.55 ^cd^	17.10 ± 0.83^bc^
	T120	3.65 ± 0.90^a^	4.09 ± 0.61^bc^	11.76 ± 0.85^d^	16.01 ± 0.53^c^

a The results are presented as mean ± SD (*n* = 3) and expressed as mg/100 g dry rice flour (mg/100 g DF). Values in each row with different letters are significantly different (*P* < 0.05). GA, gallic acid; PA, protocatechuic acid; 2,5-HA, 2,5-dihydroxybenzoic, *p*-HA, *p*-hydroxybenzoic acid; VA, vanillic acid; SA, syringic acid; *p*-CA, *p*-coumaric acid; FA, ferulic acid; SNA, sinapic acid; IFA, isoferulic acid; TR, trace amount; ND, not detected. Total, total phenolic fraction: a sum of free/conjugated and bound phenolic fractions; BR, black rice; T90, black rice extruded at 90 °C; T100, black rice extruded at 100 °C; T110, black rice extruded at 110 °C; T120, black rice extruded at 120 °C.

For the soluble-free phenolic acid fraction, the GA, PA, VA, *p*-CA and IFA contents presented similar changes with increasing extrusion temperature; they significantly (*P* < 0.05) increased from 4.34, 7.30, 11.41, 1.68 and 1.26 mg/100 g DF to 8.47, 10.99, 25.08, 2.01 and 3.65 mg/100 g DF, respectively. However, the 2,5-HA, FA and SNA contents of the extrudates at 90 °C were obviously lower than those of black rice; they decreased from 10.34, 6.62 and 2.36 mg/100 g DF to 4.53, 4.47 and 1.33 mg/100 g DF, respectively. As the extrusion temperature gradually increased, the 2,5-HA and SNA contents obviously increased to 7.08 and 2.62 mg/100 g DF, respectively. In addition, the *p*-HA contents retained similar levels in black rice and all the treated samples. SA was detected in trace amounts. Overall, the soluble-free phenolic contents significantly increased. This is attributed to the higher temperature and strong shear force of extrusion, which can partially break down the ester bonds between phenolics and cell walls or components of complex structures such as cellulose, lignin and proteins. Rochín-Medina *et al.* also reported that extrusion of whole cereals can release bound phenolics due to breaking of conjugated moieties.^25^ Therefore, some soluble-conjugated and insoluble-bound phenolics were released as soluble-free phenolics. Similar increases were described in previous reports.^10,26^

For the soluble-conjugated phenolic acid fraction, a significant (*P* < 0.05) decrease was observed when black rice was treated by extrusion. However, with increasing extrusion temperature, the soluble-conjugated phenolic acid content remained at a constant level. Interestingly, the trend of the change in insoluble-bound phenolic acid content was opposite to that of soluble-conjugated phenolic acid. That is to say, extrusion resulted in an increase of the insoluble-bound phenolic acid content, while similar levels were maintained at different extrusion temperatures. This indicates that some soluble-free and soluble-conjugated phenolics can transform into insoluble-bound phenolics; these are not linked to cell walls through ester bounds but are combined with a complex formed between lipids, proteins, starch and high molecular weight compounds during the extrusion process.^27^ In addition, the insoluble-bound 2, 5-HA, SA, and SNA were not detected in black rice but were found in the extrudates of black rice. Hence, the results also confirmed that soluble-free and soluble-conjugated phenolics transformed into insoluble-bound phenolics.

For the total phenolic acid fraction, the GA and VA contents significantly (*P* < 0.05) decreased from 42.72 and 148.50 to 21.75 and 141.65 mg/100 g DF and then increased to 24.00 and 158.46 mg/100 g DF, respectively. The SNA content significantly (*P* < 0.05) decreased and then maintained a constant level. The IFA content gradually decreased from 26.74 to 16.01 mg/100 g DF. Additionally, the PA, 2,5-HA, *p*-HA, SA, *p*-CA and FA contents of the extrudates were significantly (*P* < 0.05) higher than those of black rice. However, there was no significant difference between the different extrudates with increasing extrusion temperature. Overall, extrusion can increase the content of total phenolics; with increasing extrusion temperature, the total phenolics gradually increased and reached peak values in the range from 110 °C to 120 °C. However, some studies also reported that higher extrusion temperatures (over 120 °C) resulted in the degradation of phenolic compounds due to changes in the molecular structures of the phenolic compounds,^28^ which reduced their chemical reactivity or decreased their extractability.^29^ Therefore, the choice of extrusion temperature should be regarded as a critical parameter in food processing in order to obtain extrusion products with good functional and textural properties.

### 3.3 Effects of different extrusion temperatures on total phenolic content (TPC) and antioxidant activity

Antioxidant activity is higher in food products containing polyphenols, flavonoids, anthocyanin, procyanidine and phytosterols. Changes in their contents during food processing can significantly impact the antioxidant activity of the products. In this study, the TPC and antioxidant activities (DPPH and ABTS˙^+^ radical scavenging capacities) of the soluble-free, soluble-conjugated and insoluble-bound phenolic acid fractions of black rice and all extrudates are shown in Table 2. After extrusion, the soluble-free and soluble-conjugated TPC significantly (*P* < 0.05) decreased from 75.46 and 94.72 mg GAE/100 g DF to 44.54 and 67.25 mg GAE/100 g DF, respectively, while insoluble-bound TPC significantly (*P* < 0.05) increased from 45.70 to 65.83 mg GAE/100 g DF. With increasing extrusion temperature, soluble-free TPC slightly increased to 52.45 mg GAE/100 g DF at 110 °C, while soluble-conjugated TPC maintained a constant level; insoluble-bound TPC (73.59 mg GAE/100 g DF) reached a peak value at 110 °C.

**Table 2:** Total phenolic contents and antioxidant activities of soluble-free, soluble-conjugated and insoluble-bound fractions of black rice at different extrusion temperatures^a^

	TPC (mg GAE/100 g DF)			DPPH (μM TE/100 g DF)			ABTS (μM TE/100 g DF)		
	Soluble-free	Soluble-conjugated	Insoluble-bound	Soluble-free	Soluble-conjugated	Insoluble-bound	Soluble-free	Soluble-conjugated	Insoluble-bound
BR	75.46 ± 4.60^a^	94.72 ± 4.76^a^	45.70 ± 0.49^c^	48.23 ± 3.78^a^	68.42 ± 3.75^a^	44.45 ± 0.33^c^	79.17 ± 6.01^a^	98.93 ± 14.15^a^	94.52 ± 2.99^c^
T_90_	44.54 ± 1.03^c^	67.25 ± 0.43^b^	65.83 ± 1.04^b^	27.47 ± 0.40^b^	45.39 ± 3.35^c^	59.57 ± 0.81^b^	43.90 ± 5.25^c^	55.95 ± 8.08^c^	110.79 ± 4.01^ab^
T_100_	48.46 ± 2.37^bc^	68.52 ± 1.70^b^	69.78 ± 3.90^a^	28.64 ± 0.84^b^	51.03 ± 5.87^bc^	64.33 ± 2.39^a^	35.58 ± 3.50^d^	66.89 ± 2.04^bc^	104.10 ± 3.42^b^
T_110_	52.45 ± 1.29^b^	67.67 ± 1.38^b^	73.59 ± 2.61^a^	28.61 ± 1.68^b^	57.92 ± 1.39^b^	64.10 ± 1.32^a^	58.89 ± 3.00^b^	70.65 ± 2.91^b^	119.26 ± 0.06^a^
T_120_	52.26 ± 1.69^b^	65.30 ± 3.10^b^	73.46 ± 0.04^a^	27.50 ± 1.82^b^	53.16 ± 3.31^b^	64.35 ± 1.55^a^	61.19 ± 2.74^b^	70.87 ± 1.85^b^	118.10 ± 4.15^a^

a The results are presented as mean ± SD (*n* = 3). Values in each column with different letters are significantly different (*P* < 0.05); TPC, total phenolic content; DPPH, antioxidant capacity tested by DPPH radical scavenging; ABTS, antioxidant capacity tested by ABTS˙^+^ radical scavenging; BR, black rice; T90, black rice extruded at 90 °C; T100, black rice extruded at 100 °C; T110, black rice extruded at 110 °C; T120, black rice extruded at 120 °C.

DPPH radical and ABTS cationic scavenging activities were used to measure the antioxidant activities of the extracts. Many studies have reported that the trends of antioxidant activity are generally similar to that of the TPC; such changes were also found in this work, which may be due to the high correlation between antioxidant activity and TPC.^17,30^

As shown in Table 2, BR had higher soluble-free DPPH and ABTS values than T90, and similar changes in the soluble-conjugated DPPH and ABTS values were observed. However, T90 had higher values for insoluble-bound DPPH and ABTS than BR. With increasing extrusion temperature, soluble-free DPPH maintained a similar level, while ABTS significantly (*P* < 0.05) increased from 43.90 to 61.19 μM TE/100 g DF. Meanwhile, soluble-conjugated DPPH (57.92 μM TE/100 g DF) and ABTS (70.87 μM TE/100 g DF) reached peak values in the range from 110 °C to 120 °C. Insoluble-bound DPPH had higher levels ranging from 100 °C to 120 °C and ABTS ranging from 110 °C to 120 °C. Shao *et al.* reported that PA, VA, SA and FA are associated with TPC and antioxidant activity in the soluble-conjugated fraction, while PA and FA are correlated with those in the insoluble-bound fraction.^31^ As shown in Table 1, PA, VA, SA and FA in the soluble-conjugated fraction significantly decreased after extrusion and reached a peak value in the range from 110 °C to 120 °C. PA and FA in the insoluble-bound fraction significantly increased with increasing extrusion temperature. These results were consistent with the changes in TPC and antioxidant activity in this study.

### 3.4 Correlation analysis between extrusion temperature, die pressure and different parameters (phenolic acids, TPC, DPPH and ABTS)

We assessed the effects of extrusion behavior (extrusion temperature and die pressure) on the phenolic contents and antioxidant activities of the extrusion products. The correlations between extrusion temperature, die pressure and different parameters (phenolic acids, TPC, DPPH and ABTS) are listed in Tables 3 and 4. For the phenolic acid fraction, the results showed that soluble-free phenolic acids were significantly (*P* < 0.05) positively correlated with extrusion temperature and negatively correlated with die pressure, except FA. Meanwhile, soluble-conjugated phenolic acids had no significant correlative relationship with extrusion temperature and die pressure except SA and SNA, which may be due to the fact that soluble-conjugated phenolics transformed into insoluble-bound phenolics as mentioned above. Similarly, there were no consistent correlations between extrusion temperature, die pressure and insoluble-bound phenolics except 2,5-HA and *p*-CA. VA, *p*-CA and FA were abundant among the total phenolics of the extrusion products, which was significantly (*P* < 0.05) positively correlated with extrusion temperature and negatively correlated with die pressure. It can be speculated that increasing extrusion temperature and decreasing die pressure result in increases in the soluble-free and total phenolic acid contents. For TPC and antioxidant activity (DPPH and ABTS), the soluble-free and insoluble-bound TPC were significantly (*P* < 0.05) positively correlated with extrusion temperature; however, only soluble-free TPC was significantly (*P* < 0.05) negatively correlated with die pressure. There were no significant correlative relationships between extrusion temperature, die pressure and antioxidant activity except for soluble-free and soluble-conjugated ABTS. It can be concluded that extrusion temperature and die pressure do not directly affect antioxidant activity, which may be due to variations of the physicochemical content in extrusion processing. Shao *et al.* reported that individual phenolic acids present in soluble-free, soluble-conjugated and insoluble-bound phenolic fractions exert their antioxidant activities differently. They may act individually, synergistically or antagonistically; these molecular mechanisms need to be clarified with further studies.^31^

**Table 3:** Correlation analysis between extrusion temperature and different parameters (phenolic acids, TPC, DPPH and ABTS)^a^

	Soluble-free	Soluble-conjugated	Insoluble-bound	Total
GA	0.817	0.646	0.617	0.744
PA	0.970*	−0.416	0.585	0.439
2,5-HA	0.999***	−0.055	0.934*	0.507
*p*-HA	0.984*	−0.705	0.394	−0.117
VA	0.993**	−0.128	0.902	0.817*
SA	—	−0.975*	0.713	−0.564
*p*-CA	0.927*	−0.293	0.977*	0.953*
FA	−0.849*	−0.084	0.923	0.910*
SNA	0.698	0.845*	−0.826	0.581
IFA	0.908*	0.019	−0.991	−0.896
TPC	0.937*	−0.635	0.938*	—
DPPH	0.012	0.750	0.776	—
ABTS	0.793	0.895*	0.679	—

a *, **, and *** indicate significant levels at 0.05, 0.01, and 0.001, respectively.

**Table 4:** Correlation analysis between die pressure and different parameters (phenolic acids, TPC, DPPH and ABTS)^a^

	Soluble-free	Soluble-conjugated	Insoluble-bound	Total
GA	−0.692	−0.518	−0.605	−0.617
PA	−0.924*	0.580	−0.632	−0.421
2,5-HA	−0.986**	−0.137	−0.984**	−0.665
*p*-HA	−0.950*	0.824	−0.517	0.103
VA	−0.970*	0.303	−0.848	−0.695*
SA	—	0.953*	−0.749	0.463
*p*-CA	−0.922*	0.475	−0.978*	−0.890*
FA	0.841*	0.274	−0.836	−0.815*
SNA	−0.557	−0.833*	0.903	−0.470
IFA	−0.809*	−0.216	0.948	0.792
TPC	−0.874*	0.765	−0.873	—
DPPH	0.169	−0.659	−0.637	—
ABTS	−0.868*	−0.792	−0.763	—

a * and ** indicate significant levels at 0.05 and 0.01, respectively.

### 3.5 Effects of different extrusion temperatures on anthocyanin composition

**Fig. 2:**
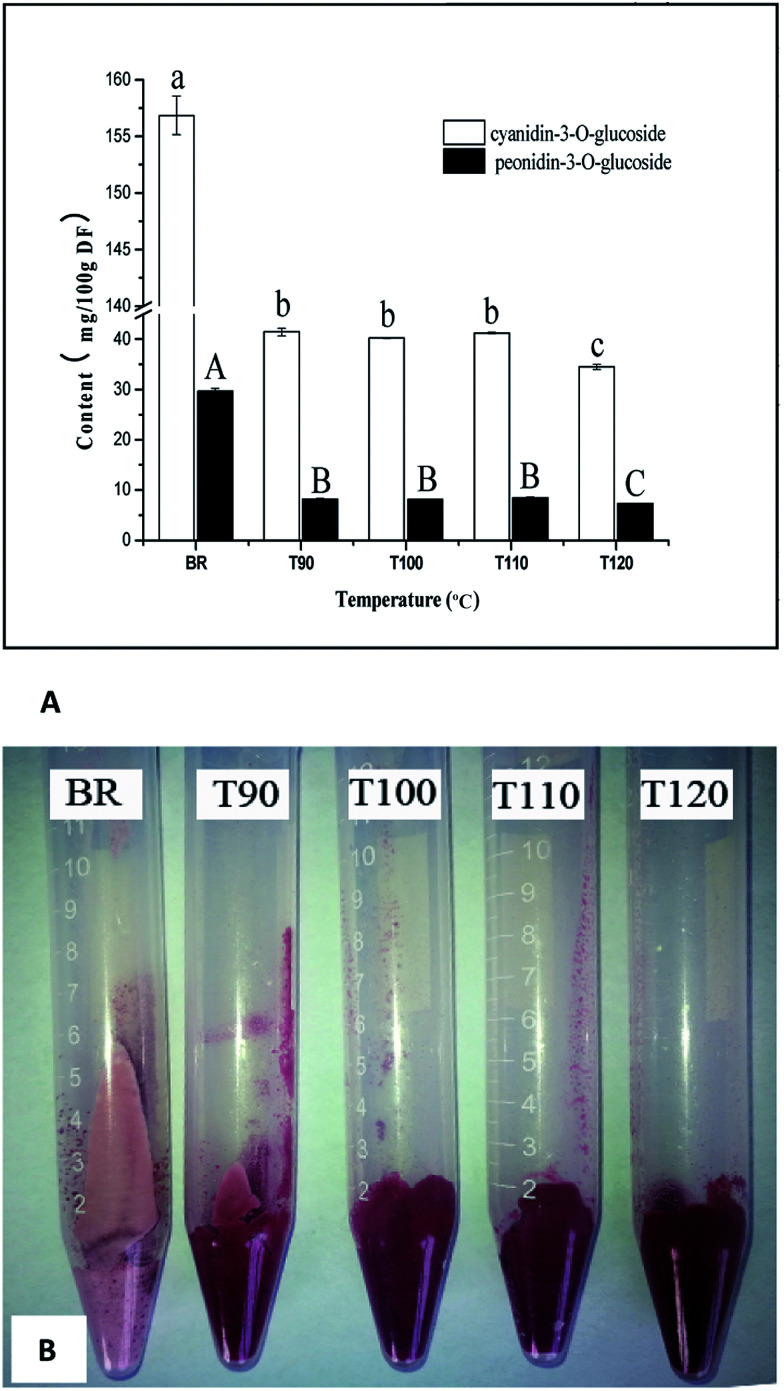
(A) Effects of different extrusion temperatures on cyanidin-3-*O*-glucoside and pennidin-3-*O*-glucoside. (B) Image of the residues after extraction of anthocyanins.*Different lowercase and capital letters above the bars indicate significant differences (*P* < 0.05). BR, black rice; T90, black rice extruded at 90 °C; T100, black rice extruded at 100 °C; T110, black rice extruded at 110 °C; T120, black rice extruded at 120 °C.

The anthocyanin compounds in black rice are mainly cyandin-3-*O*-glucoside and peonidin-3-*O*-glucoside, which has been extensively reported.^32^ Similarly, cyandin-3-*O*-glucoside and peonidin-3-*O*-glucoside were also detected in this work. The changes in anthocyanin content at different extrusion temperatures are presented in Fig. 2A. Significant decreases in cyandin-3-*O*-glucoside and peonidin-3-*O*-glucoside content were observed when black rice was subjected to extrusion treatment at 90 °C. The increases in extrusion temperature ranging from 90 °C to 110 °C did not result in a continuous decline of the anthocyanin content, and both the cyandin-3-*O*-glucoside and peonidin-3-*O*-glucoside contents remained at a constant level. Khanal *et al.* reported similar results, where extrusion resulted in the loss of anthocyanin in blueberry;^33^ however, changes in extrusion temperature had no significant effects on the total anthocyanin content. The reason for this is not clear and may be due to the fact that extrusion produced a special complex which combined lipids, proteins, starch and some high molecular weight compounds, including the abovementioned phenolic acid, anthocyanin. Martínez, Rosell and Gómez reported that extrusion affected starch functionality due to interactions between starch and the non-starch components of flours.^34^ Therefore, it is possible that the textural changes can preserve more nutritional components in the interacting complex and prevent their degradation and loss during extrusion processing.

In order to investigate the reason for this change, the residue after extraction of anthocyanins was photographed to analyze the difference in colour. As shown in Fig. 2B, the colour of the extrudate residue after extraction was redder than that of black rice. These results indicated that partial anthocyanin was retained in the residue after extraction and could not be completely extracted with aqueous organic extracts. However, a higher extrusion temperature (120 °C) resulted in a slight loss of anthocyanin. These results were also consistent with previous studies.^35^

### 3.6 Effects of different extrusion temperatures on sterol composition

Sterols exist mainly in plant cell walls and membranes. Many studies have indicated that natural dietary sterols have prominent effects on body cholesterol metabolism,^36^ which provides potential protection from coronary heart disease. Jiang and Wang showed that sitosterol, campesterol and stigmasterol are the major sterols in rice; cycloartenol and 24-methylenecycloarta-*n*-3β-ol were identified to be present in considerable amounts.^37^ In this study, six sterols were also identified in the free and bound sterol fractions of black rice and its extrudates. Among the identified sterols, the predominant sterol is β-sitosterol, followed by stigmasterol, campesterol and Δ-5-avenasterol. Cycloartenol and 24-methyl-enecycloartan-3β-ol are also present in small amounts in all samples. This result is consistent with previous findings in walnuts.^38^

**Fig. 3:**
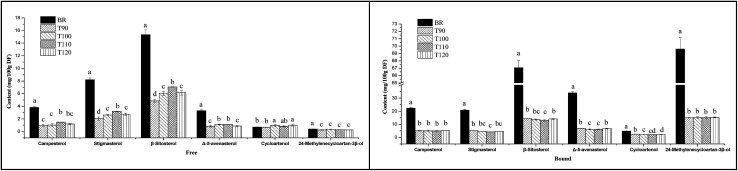
Effects of different extrusion temperatures on the contents of six sterols in black rice. The unit was mg/100 g DF. Free, free sterol fraction; Bound, bound sterol fraction; BR, black rice; T90, black rice extruded at 90 °C; T100, black rice extruded at 100 °C; T110, black rice extruded at 110 °C; T120, black rice extruded at 120 °C. *Different lowercase letters above bars indicate significant differences (*P* < 0.05).

**Fig. 4:**
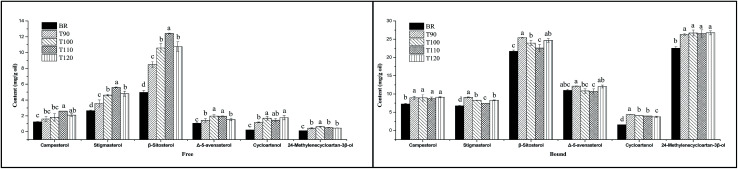
Effects of different extrusion temperatures on the contents of six sterols in black rice. The unit was mg g^−1^ oil. Free, free sterol fraction; bound, bound sterol fraction; BR, black rice; T90, black rice extruded at 90 °C; T100, black rice extruded at 100 °C; T110, black rice extruded at 110 °C; T120, black rice extruded at 120 °C. *Different lowercase letters above the bars are significantly different, respectively (*P* < 0.05).

In addition, two units used in many previous studies were applied to express the sterol content in order to analyse the effects of different extrusion temperatures on sterol composition. As shown in Fig. 3, when the sterol content was expressed as mg/100 g DF, the free and bound sterol contents of the extrudates were significantly lower than that of BR. However, with increasing extrusion temperature, the free sterol content significantly increased from 90 °C to 110 °C and decreased at 120 °C. Especially, the free campesterol, stigmasterol and β-sitosterol contents reached peak values at 110 °C. For bound sterol content, high temperature resulted in a decrease of sterol content, and the bound campesterol, stigmasterol and β-sitosterol contents had lower levels at 110 °C. When the sterol content was expressed as mg g^−1^ oil, as shown in Fig. 4, the free and bound sterol contents of the extrudates were significantly higher than those of BR. With increasing extrusion temperature, the free sterol content significantly increased from 90 °C to 110 °C and decreased at 120 °C, and the bound sterol content significantly decreased from 90 °C to 110 °C, except campesterol and 24-methylenecycloartan-3β-ol.

## 4. Conclusions

In summary, different extrusion temperatures result in changes in the extrusion behavior, phenolic acid contents, antioxidant activities, anthocyanins and sterol contents of extrusion products of black rice. The peak value of the expansion ratio of the extrudates can be attributed to the interaction between extrusion temperature and die pressure. Similarly, extrusion temperature and die pressure also have significant effects on the physicochemical properties of the extrusion products. With increasing extrusion temperature and decreasing die pressure, the soluble-free and total phenolic acid contents gradually increased, while portions of the soluble-free and soluble-conjugated phenolic acids could transform into insoluble-bound phenolic acids. TPC and antioxidant activity had higher levels in the range from 110 °C to 120 °C. Because anthocyanin cannot be completely extracted with aqueous organic extracts, the results show that the anthocyanin contents of the extrudates are significantly lower than that of BR. In addition, due to the interaction between the extrusion temperature and die pressure, the free sterol content has a higher level at 110 °C and the bound sterol content has a lower level at 110 °C. The aim of this study is to provide new information for rice producers and food scientists who are seeking to obtain rice products which are rich in health-promoting phytochemicals, and to understand the changes in phytochemicals and antioxidant properties in black rice and its extrudates at different extrusion temperatures.

## Conflicts of interest

The authors declare that there are no conflicts of interest.

## Supplementary Material

RA-008-C7RA13329D-s001
