# Supplementary material for: Effects of different extrusion temperatures on extrusion behavior, phenolic acids, antioxidant activity, anthocyanins and phytosterols of black rice
Source: RSC Adv. 2018 Feb 14;8(13):7123–32. doi: 10.1039/c7ra13329d (PMC9078409; doi:10.1039/c7ra13329d)
Supplement: RA-008-C7RA13329D-s001 [file RA-008-C7RA13329D-s001.pdf]

Electronic Supplementary Material (ESI) for RSC Advances.
This journal is © The Royal Society of Chemistry 2018

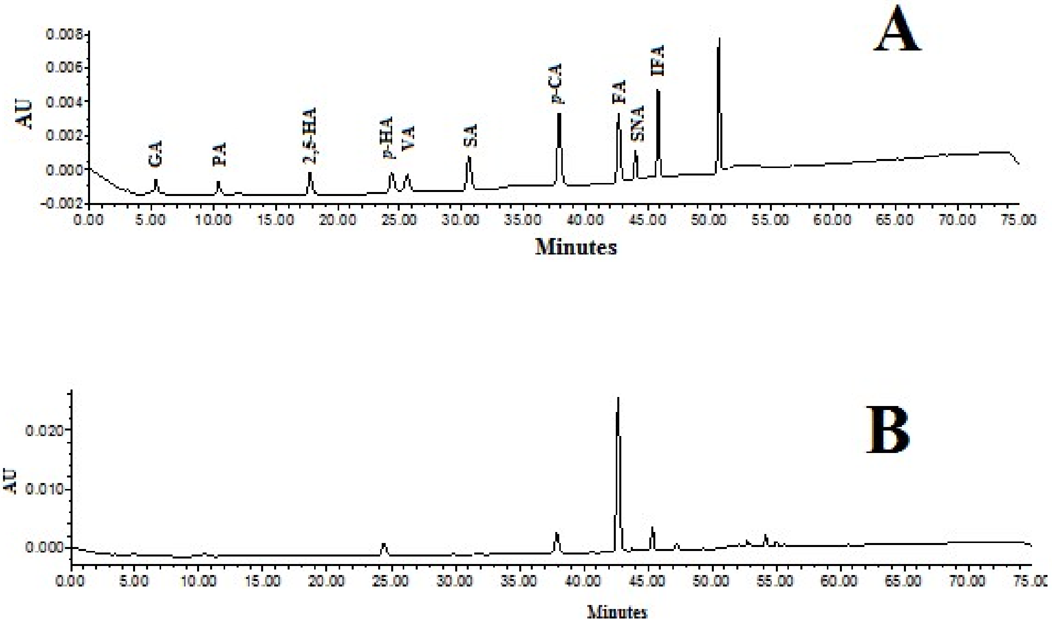


Fig 1 the HPLC chromatogram of phenolic acid standards (A) and sample (B)

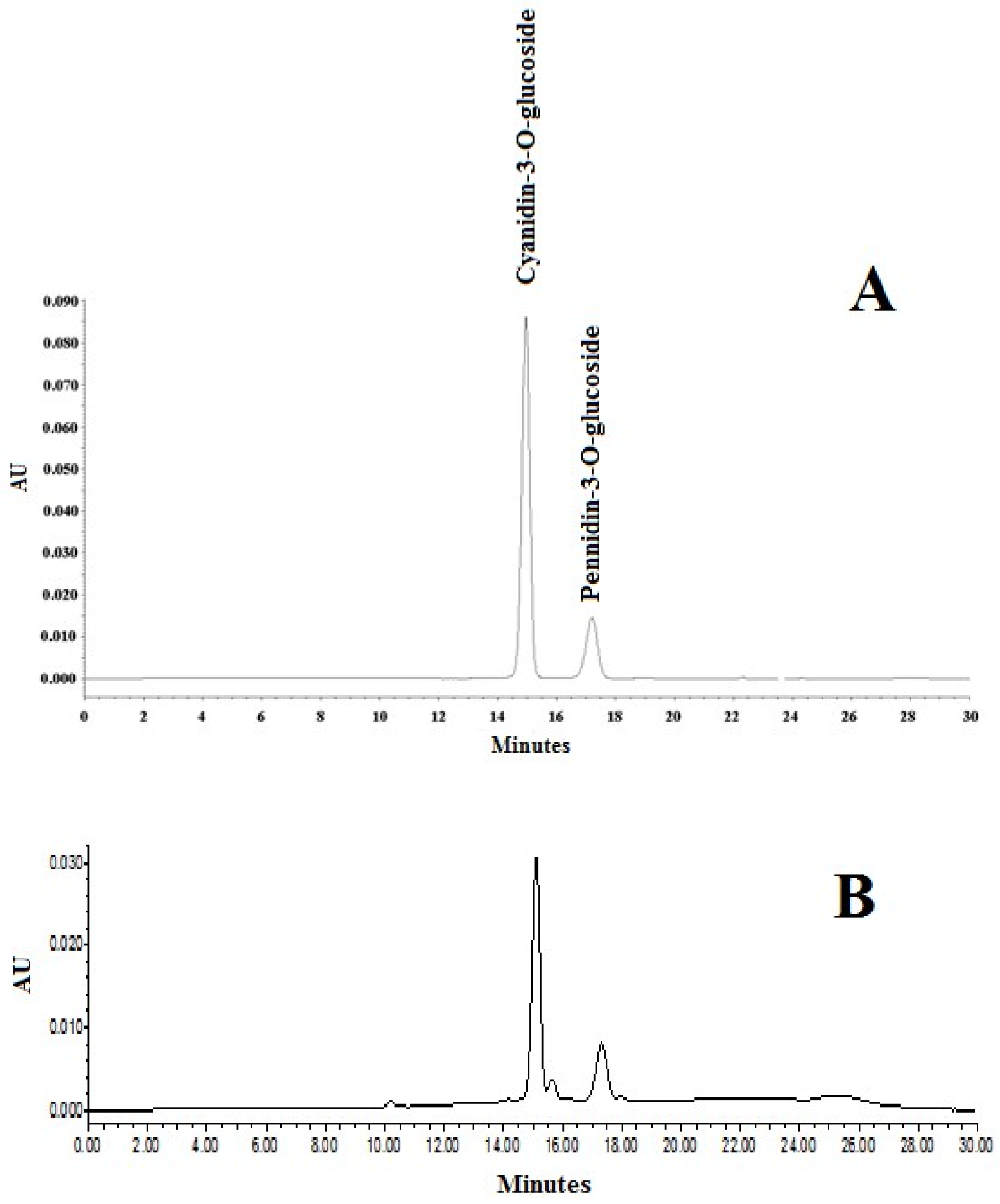


Fig 2 the HPLC chromatogram of anthocyanin standards (A) and sample (B)

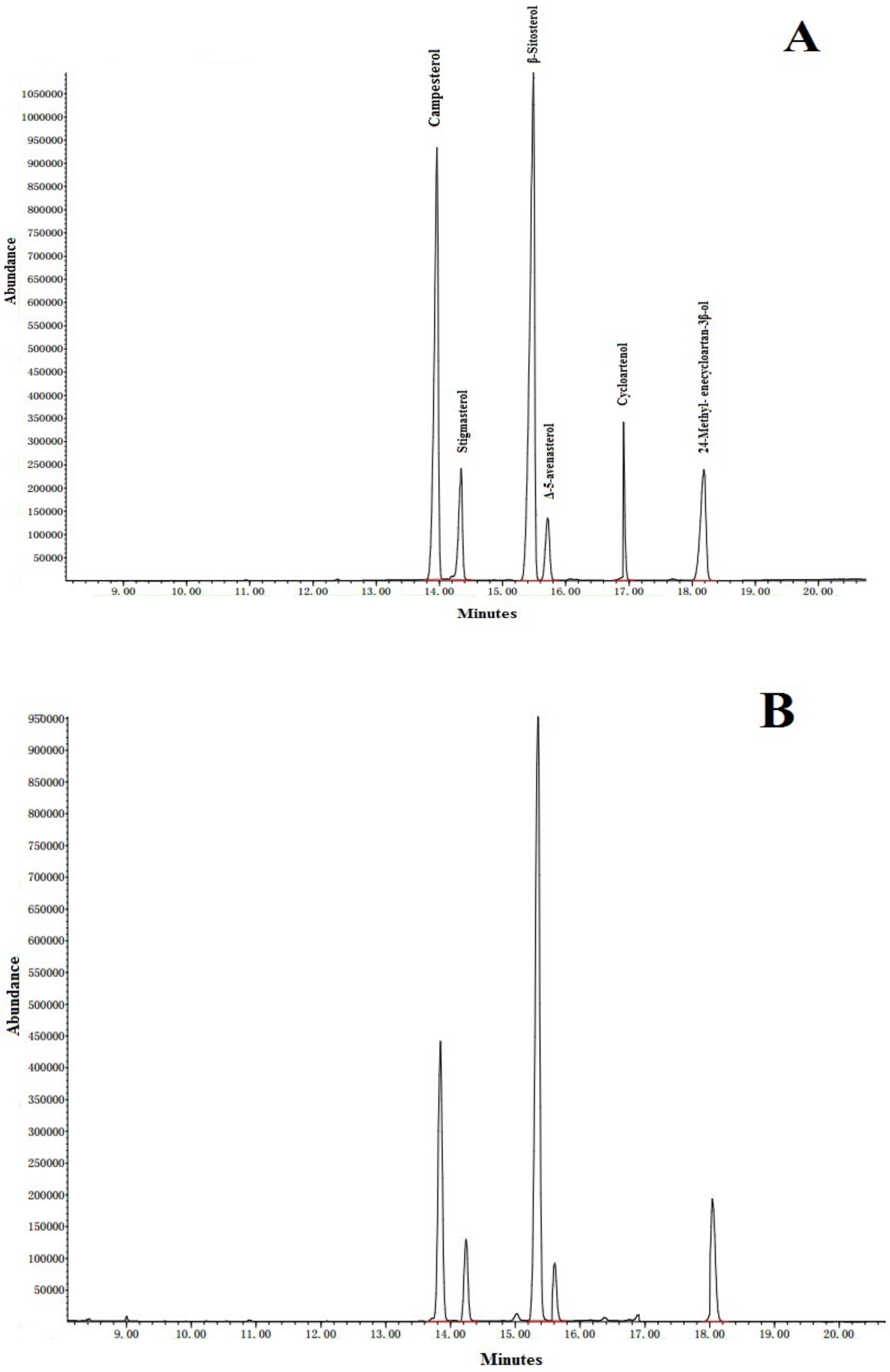


Fig 3 the GC-MS chromatogram of sterol standards (A) and sample (B)
